# Nationwide registry of sepsis patients in Japan focused on disseminated intravascular coagulation 2011–2013

**DOI:** 10.1038/sdata.2018.243

**Published:** 2018-12-11

**Authors:** Mineji Hayakawa, Kazuma Yamakawa, Shinjiro Saito, Shigehiko Uchino, Daisuke Kudo, Yusuke Iizuka, Masamitsu Sanui, Kohei Takimoto, Toshihiko Mayumi

**Affiliations:** 1 Emergency and Critical Care Center, Hokkaido University Hospital, Sapporo, 060-8648 Japan; 2 Division of Trauma and Surgical Critical Care, Osaka General Medical Center, Osaka, 565-0871 Japan; 3 Intensive Care Unit, Department of Anesthesiology, Jikei University School of Medicine, Tokyo, 105-8471 Japan; 4 Division of Emergency and Critical Care Medicine, Tohoku University Graduate School of Medicine, Sendai, 980-8574 Japan; 5 Department of Anesthesiology and Critical Care Medicine, Jichi Medical University Saitama Medical Center, Saitama, 330-8503 Japan; 6 Department of Critical Care, Shonan Kamakura General Hospital, Kamakura, Japan; 7 Department of Anesthesiology and Intensive Care Medicine, Osaka University Graduate School of Medicine, Osaka, 565-0871 Japan; 8 Department of Emergency Medicine, University of Occupational and Environmental Health, Kitakyuusyu, 807-8556 Japan

**Keywords:** Therapeutics, Haematological diseases, Infection

## Abstract

Sepsis is a syndrome with physiologic, pathologic, and biochemical abnormalities induced by infection. Sepsis can induce the dysregulation of systemic coagulation and fibrinolytic systems, resulting in disseminated intravascular coagulation (DIC), which is associated with a high mortality rate. Although there is no international consensus on available treatments for sepsis-induced DIC, DIC diagnosis and treatment are commonly performed in Japanese clinical settings. Therefore, clinical data related to sepsis-induced DIC diagnosis and treatment can be obtained from Japanese clinical settings. We performed a retrospective nationwide observational study (Japan Septic Disseminated Intravascular Coagulation [J-SEPTIC DIC] study) to collect data regarding characteristics of sepsis patients in Japan, with a focus on coagulofibrinolytic dysregulation and DIC treatment received by each patient. The J-SEPTIC DIC study collected information for a total of 3,195 patients with severe sepsis and septic shock and is the largest data set in Japan on DIC diagnosis and treatment in clinical settings.

## Background & Summary

Sepsis is a syndrome with physiologic, pathologic, and biochemical abnormalities induced by infection and resulting in life-threatening organ dysfunction^1,2^. A global epidemiological report estimated that 31.5 million people are affected by sepsis and 19.4 million people are affected by severe sepsis each year, with a potential 5.3 million deaths worldwide from sepsis each year^3^.

Disseminated intravascular coagulation (DIC) is induced by the dysregulation of systemic coagulation and fibrinolytic systems in sepsis and septic shock^2,4,5^. Sepsis-induced DIC causes the development of microthrombi, which cause tissue hypoperfusion and result in multiple organ failure; sepsis-induced DIC is thus associated with a high mortality rate^2,4,5^. However, because appropriate treatments for sepsis-induced DIC have not been widely studied, there is no international consensus on available treatments for sepsis-induced DIC^6,7^, and in many countries specific treatment for sepsis-induced DIC in clinical settings is not provided^8^. On the other hand, in Japanese clinical settings, DIC diagnosis using scoring systems are generalized in sepsis management^9^. Furthermore, recombinant thrombomodulin, antithrombin and other anticoagulants are approved as DIC treatment drugs and are frequently used in patients with sepsis-induced DIC^10,11^. Therefore, clinical data related to the treatment of sepsis-induced DIC can be collected from clinical settings in Japan.

We performed a retrospective nationwide observational study (Japan Septic Disseminated Intravascular Coagulation [J-SEPTIC DIC] study), which collected data on the characteristics of sepsis patients with a focus on coagulation dysregulation and DIC treatments. Previous studies on DIC treatment have been conducted using the J-SEPTIC DIC data set^12–20^.

## Methods

**Figure 1:**
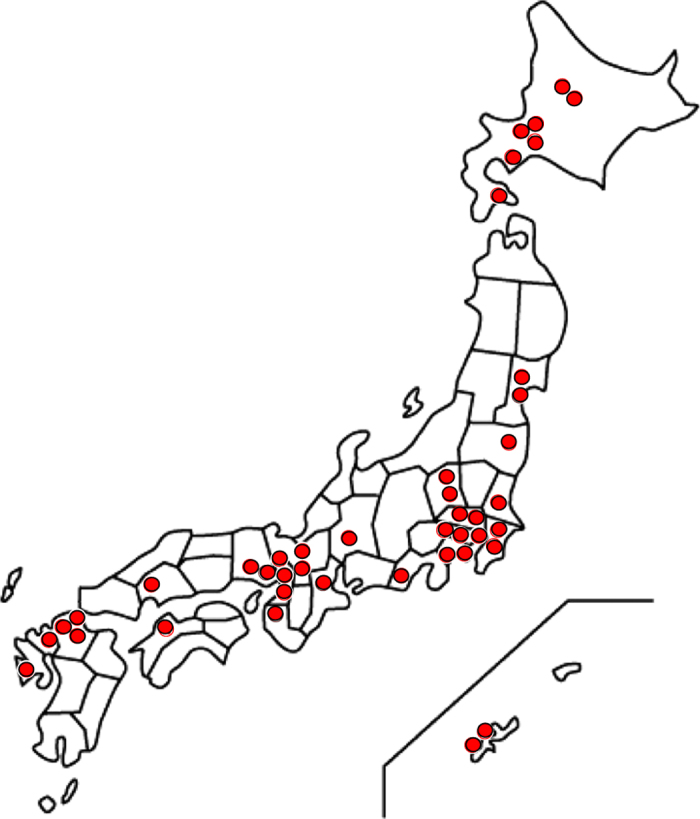
Locations of the participating institutions. Participating institutions for this data set were 42 intensive care units from 40 institutions across Japan.

The J-SEPTIC DIC study was conducted in 42 intensive care units (ICUs) of 40 institutions throughout Japan (Table 1 and Fig. 1) and was approved by the institutional review boards of each hospital. The boards waived the requirement for informed consent, due to the retrospective design.

We retrospectively reviewed data of consecutive patients who were admitted to the ICUs of participating institutions to be treated for severe sepsis or septic shock between January 2011 and December 2013. Severe sepsis and septic shock were defined based on the International Sepsis Definitions Conference criteria^21^. We excluded patients who were &lt; 16 years old, or patients who developed severe sepsis or septic shock after their ICU admission.

The following data were collected: age; sex; body weight; admission route to the ICU; pre-existing organ dysfunction; pre-existing hemostatic disorder; Acute Physiology and Chronic Health Evaluation (APACHE) II score;^22^ Sequential Organ Failure Assessment (SOFA) score^23^ (days 1, 3, and 7); systemic inflammatory response syndrome (SIRS) score^24^ (days 1, 3, and 7); primary infection site; blood culture results; microorganisms responsible for the sepsis; daily results from laboratory tests during the first week after ICU admission; lactate levels (days 1, 3, and 7); administration of drugs, including immunoglobulins, and low-dose steroids, during the first week after ICU admission; therapeutic interventions, including surgical interventions at the infection site, renal replacement therapy, renal replacement therapy for non-renal indications, polymyxin B direct hemoperfusion, extracorporeal membrane oxygenation, and intra-aortic balloon pumping, during the first week after ICU admission; and outcomes in the hospital.

The following data related to DIC diagnosis and treatment were also collected: systemic inflammatory response syndrome score; daily results from laboratory tests, which included platelets counts, prothrombin time/international normalized ratio, fibrinogen level, and antithrombin activity; D-dimer levels; fibrin/fibrinogen degradation product levels during the first week after ICU admission; administration of anti-DIC drugs, which included antithrombin, recombinant thrombomodulin, protease inhibitors and heparinoids, and other anticoagulants during the first week after ICU admission; and transfusion amounts and bleeding complications during the first week after ICU admission.

Finally, the following data related to the institutions and ICUs were collected: characteristics of institutions and ICUs (general ICU or emergency ICU); management policy of the ICU (closed or open); number of beds in the ICU; reagents of fibrin/fibrinogen degradation products and D-dimer measurements.

Several analyses have already been conducted and studies have been published using this data set^11–16,18–20,25–28^.

## Data Records

A single data set resulted from the present study. This data set contains information of the 3,195 patients with severe sepsis or septic shock in 42 ICUs over 3 years. The information of the institution and the ICU where each patient was admitted is described in the same line as the patient’s information (Data Citation 1). Blanks in the data set indicate missing data. In the present study, all laboratory results were measured according to clinical necessity. Therefore, many missing data were included in the data set. Furthermore, some variables were not available due to death or discharge.

Detailed information on variable specifications is included in a Read_me file (Data Citation 1).

## Technical Validation

The present study was a retrospective design. Information of eligible patients was collected in each participating institute and reported to the principal institute (Hokkaido University Hospital) by one investigator per institution. Collected data were assessed by expert emergency and critical care physicians; if outliers in each variable and contradictions within data were detected, data were validated with each investigator in each hospital. The outliers and contradictions were judged by the expert emergency and critical care physicians. Data were finalized and fully anonymized on September 8, 2015.

## Additional information

**How to cite this article**: Hayakawa, M. *et al.* Nationwide registry of sepsis patients in Japan focused on disseminated intravascular coagulation 2011–2013. *Sci. Data*. 5:180243 doi:10.1038/sdata.2018.243 (2018).

**Publisher’s note**: Springer Nature remains neutral with regard to jurisdictional claims in published maps and institutional affiliations.

## Supplementary Material



## Figures and Tables

**Table 1:** List of participating institutions.

**Institutions**
Akashi City Hospital
Asahikawa Medical University
Asahikawa Red Cross Hospital
Ehime University Hospital
Fukuoka University Hospital
Gifu University Hospital
Graduate School of Medicine, University of the Ryukyus
Gunma University
Hakodate Municipal Hospital
Hokkaido University Hospital
Hyogo College of Medicine
Ibaraki Prefectural Central Hospital
JA Hiroshima General Hospital
Japan Red Cross Maebashi Hospital
Jichi Medical University Saitama Medical Center
Jikei University School of Medicine
Kameda Medical Center
KKR Sapporo Medical Center
Kyoto First Red-Cross Hospital
Kyushu University Hospital
Mie University Hospital
Nagasaki University Hospital
Nihon University School of Medicine
Nippon Medical School Chiba Hokusoh Hospital
Ohta General Hospital Foundation Ohta Nishinouchi Hospital
Osaka General Medical Center
Osaka University Hospital
Saga University Hospital
Saiseikai Yokohamasi Tobu Hospital
Saitama Red Cross Hospital
Sapporo City General Hospital
Seirei Mikatahara General Hospital
Sendai City Hospital
Shonan Kamakura General Hospital
Steel Memorial Muroran Hospital
Tohoku University Hospital
Tokyo Medical University, Hachioji Medical Center
Tomishiro Central Hospital
University of Occupational and Environmental Health Hospital
Wakayama Medical University Hospital
